# What are you looking at? Gaze following with and without target objects in ASD and typical development

**DOI:** 10.1177/13623613211061940

**Published:** 2021-12-14

**Authors:** Emilia Thorup, Pär Nyström, Sven Bölte, Terje Falck-Ytter

**Affiliations:** 1 Uppsala University, Sweden; 2 Lund University, Sweden; 3 Karolinska Institutet, Sweden; 4 Stockholm Health Care Services, Region Stockholm, Sweden; 5 Curtin University, Australia; 6 Swedish Collegium for Advanced Study, Sweden

**Keywords:** attention, autism spectrum disorders, development, eyetracking, gaze following, joint attention, social cognition and social behavior

## Abstract

Children with autism spectrum disorder (ASD) display difficulties with response to joint attention in natural settings but often perform comparably to typically developing (TD) children in experimental studies of gaze following. Previous work comparing infants at elevated likelihood for ASD versus TD infants has manipulated aspects of the gaze cueing stimulus (e.g. eyes only versus head and eyes together), but the role the peripheral object being attended to is not known. In this study of infants at elevated likelihood of ASD (*N* = 97) and TD infants (*N* = 29), we manipulated whether or not a target object was present in the cued area. Performance was assessed at 10, 14, and 18 months, and diagnostic assessment was conducted at age 3 years. The results showed that although infants with later ASD followed gaze to the same extent as TD infants in all conditions, they displayed faster latencies back to the model’s face when (and only when) a peripheral object was absent. These subtle atypicalities in the gaze behaviors directly after gaze following may implicate a different appreciation of the communicative situation in infants with later ASD, despite their ostensively typical gaze following ability.

**Lay abstract:**

During the first year of life, infants start to align their attention with that of other people. This ability is called joint attention and facilitates social learning and language development. Although children with autism spectrum disorder (ASD) are known to engage less in joint attention compared to other children, several experimental studies have shown that they follow other’s gaze (a requirement for visual joint attention) to the same extent as other children. In this study, infants’ eye movements were measured at age 10, 14, and 18 months while watching another person look in a certain direction. A target object was either present or absent in the direction of the other person’s gaze. Some of the infants were at elevated likelihood of ASD, due to having an older autistic sibling. At age 3 years, infants were assessed for a diagnosis of ASD. Results showed that infants who met diagnostic criteria at 3 years followed gaze to the same extent as other infants. However, they then looked back at the model faster than typically developing infants *when no target object was present*. When a target object was present, there was no difference between groups. These results may be in line with the view that directly after gaze following, infants with later ASD are less influenced by other people’s gaze when processing the common attentional focus. The study adds to our understanding of both the similarities and differences in looking behaviors between infants who later receive an ASD diagnosis and other infants.

Joint attention (JA)—the sharing of attention between two individuals toward a common object (Bruner, 1975; Scaife & Bruner, 1975)—is a prerequisite for many socio-cognitive functions, including language and social learning (e.g. Morales et al., 2000; Van Hecke et al., 2012). Joint attention is also known as an area of challenge in autism spectrum disorder (ASD; e.g. Charman, 2003; Mundy, 2016). As subtle early JA alterations may have cascading effects on later development (e.g. Mundy et al., 2009), identifying early JA differences is an important step in the pursuit toward early intervention.

Response to joint attention is often operationalized as gaze following. Experimental studies of gaze following commonly entail a video of a person who turns the head in the direction of one of two target objects, usually located in close proximity to the person. In such a setting, typically developing (TD) infants usually follow the person’s gaze to the target object from around 3 to 4 months of age (Del Bianco et al., 2019; D’Entremont, 2000; D’Entremont et al., 1997; Gredebäck et al., 2010). Although less gaze following in autistic children or infants at elevated likelihood of ASD is often reported in naturalistic settings (e.g. Dawson et al., 2004), a number of studies have suggested that these children may be less challenged in a clear and highly controlled setting such as the one described above (e.g. Akechi et al., 2011; Bedford et al., 2012; Falck-Ytter et al., 2015; Gliga et al., 2012; Parsons et al., 2019). This discrepancy between real-life and laboratory renders it an important task for experimental studies to parcel out which aspects of gaze following that may be atypical and—equally important—typical, in early ASD. We have previously aimed to bridge the gap between naturalistic and experimental settings by using eye tracking during live interaction (as opposed to more common video-based eye tracking; Nyström et al., 2017; Nyström et al., 2019; Thorup et al., 2016, 2018). In this study, we use live interaction eye tracking with the aim to test the hypothesis that seeing another person look at an area with no present target object influences attention to that area more in TD infants than in infants later diagnosed with ASD.

Although ASD is still rarely diagnosed before the age of 2–3 years in most countries (Landa, 2008), knowledge about early development has increased markedly during the past 10–15 years, due to the increased use of prospective studies. Such studies often follow younger siblings of children with ASD from an early age, as 7%–20% of them are expected to later receive a diagnosis of ASD (Gronborg et al., 2013; Messinger et al., 2015). Studies of infant siblings are instrumental in the search for early diagnostic markers (Zwaigenbaum et al., 2013). This in turn, is crucial for the development of early intervention, which may be more efficient compared to later-initiated programs (Koegel et al., 2014). In one of our previous studies (Nyström et al., 2019), we followed younger siblings of autistic children and TD comparison infants, assessing various aspects of visual JA. That study focused on two main types of visual JA, namely gaze following and initiation of JA. The results showed that infants later diagnosed with ASD initiated JA to a lower degree than other infants at 10 months and that this was followed by a developmental trajectory that deviated from what was seen in typical development. Gaze following however, did not distinguish infants in terms of later diagnosis. As the previous study (Nyström et al., 2019) focused on both initiation and response to JA (and the difference between them), we included only two standard conditions in the gaze following analysis; one in which the model turned the entire head (Eyes and Head condition) in the direction of the target and one in which the model kept the head still and only used the eyes to gaze at the target (Eyes Only condition). The results showed that all infants tended to display higher gaze following accuracy in the Eyes and Head condition compared to the Eyes Only condition, but that this pattern was similar across groups (no group by condition interaction effect). In this study, we reanalyzed data from these two gaze following conditions, but also incorporated data from a third condition not included in the previous analysis. In the novel third condition, the model turned the head to look at an empty hole (from where puppets appeared in the other two conditions). We will hereafter refer to this condition as the Eyes and Head—No Object condition and to the previously described condition with head movement as the Eyes and Head—Object Present condition. Rather than probing the role of variations in the *gaze cue* (Eyes and Head—Object Present condition vs Eyes Only condition) as in our previous report, this analysis aimed to evaluate potential group differences related to the manipulations of the *gaze target* (what the infant’s interlocutor looked at). That is, we were interested in whether the absence of target objects may affect gaze following and related looking behaviors to a higher degree in infants who later receive an ASD diagnosis.

## Gaze following and object processing

As noted, studies have shown that gaze following provides a foundation for learning and development (e.g. Morales et al., 2000; Van Hecke et al., 2012). This implies that following another’s gaze to an object may affect subsequent processing of that object. Therefore, in addition to measuring gaze following accuracy, investigating other looking behaviors occurring while the model is attentionally engaged with the target, can also be informative. Moreover, a number of studies have indicated that measures such as looking duration may be more sensitive to detect group differences pertaining to ASD status than the accuracy measure (e.g. Bedford et al., 2012; Falck-Ytter et al., 2015; Freeth et al., 2010). In this study, possible group differences between infants later diagnosed with ASD and TD infants in terms of looking behaviors occurring in conjunction with successful gaze following (looking durations and latencies, see below for details) were thus investigated.

## Gaze following without target objects

To our knowledge, no previous study of infants at elevated likelihood for ASD—or for that matter, older and already diagnosed children—has investigated gaze following without target objects. Also when it comes to TD, only a few studies have assessed gaze following to areas without visible target objects. One study (Csibra & Volein, 2008) found that 8–10 months old TD infants looked longer at an empty area if another person had previously looked in that direction, compared to if the other had not looked in the direction of the empty space. Another study found that when an adult looked at an area not visible to the infant (i.e. blocked by a barrier), 12-month olds tended to walk or crawl to the area where the adult was looking (Moll & Tomasello, 2004). Together, these studies suggest that when TD infants see somebody look in a certain direction, this increases their attention to that area even if no target object is present. Children with ASD are known to display difficulties with mentalizing (e.g. Baron-Cohen, 1995; Happé, 2015), and their attention allocation may generally be less affected by others’ actions compared to TD children. In this study, we therefore tested the hypothesis that seeing another person look at an area without a present target object does not evoke an attentional heightening to that area in infants later diagnosed with ASD to the same degree as it does for TD infants.

## Aims and hypotheses

Our main measure of interest was the duration of time that infants spent looking at the target area while the model was attentionally engaged with it. We expected a group by condition interaction effect, in which infants with later ASD would spend less time than TD infants looking at the target area in the Eyes and Head−No Object condition, but not in the Eyes and Head–Object Present condition (which is identical to the Eyes and Head-No Object condition in all aspects but the presence of target objects). In addition to comparing looking time at the target area, we also compared looking time at the model’s face, as well as latencies to look back at the model after following gaze to the target (looking back at the person delivering the gaze cue has been suggested to be a key aspect of the jointness of the interaction; Siposova & Carpenter, 2019). If, as hypothesized, infants with later ASD would spend less time looking at the target in the Eyes and Head-No Object condition, one could in principle expect this group to display faster looks back to the model and longer looking time at the model in this condition as well. However, previous research (e.g. Chawarska et al., 2010, 2013; Dawson et al., 2004) indicates reduced social attention in ASD, rendering these predictions less straight forward. Hence, no directed hypotheses were formulated regarding the latter measures (looking time at face and latency to look back at face). We also did not include any directed hypothesis regarding gaze following accuracy related to the Eyes and Head-No Object condition. On one hand, it is possible that diminished social interest would render infants later diagnosed with ASD less likely to follow the model’s gaze when no target objects are present (i.e. the gaze cue may not be “enough” when it is not directed at a salient target), thus resulting in relatively lower accuracy in the Eyes and Head-No Object condition compared to the Eyes and Head-Object Present condition. On the other hand, a previous study has indicated that manipulating the interest level of target objects does not affect gaze following accuracy in either young children with TD or ASD (Thorup et al., 2017), suggesting that accuracy may not be sensitive to aspects related to the target. Performance was assessed at 10, 14, and 18 months, and developmental patterns were investigated for all measures, again with no directed hypotheses.

## Methods

### Participants

The final sample consisted of 126 infants, but note that *N*s vary between analyses as not all infants contributed data at all measurement points (see Table 1). Of these, 22 were infants at elevated likelihood of ASD who met *DSM*-5 criteria for ASD at age 3 (EL-ASD); 75 were infants at elevated likelihood who did not meet *DSM*-5 criteria for ASD at age three (EL-no-ASD); and 29 were TD comparison infants. One additional infant from the comparison group was excluded due to receiving an ASD diagnosis. Five additional infants (4 EL-no-ASD, 1 TD) were excluded due to not producing enough valid data (see Analysis). The sample partially overlapped with Nyström et al. (2019), with 98 infants (26 TD; 50 EL-no-ASD; 22 EL-ASD) contributing to both studies. EL infants were recruited through advertisement, the project’s website and clinical units. All EL infants had at least one older full sibling with a community diagnosis of ASD (verified via inspection of medical records). TD infants were recruited from live birth records and had at least one TD older full sibling, and no first or second degree relatives with ASD. Infants from both groups came predominantly from the larger Stockholm metropolitan area. Most families were of Swedish origin, but 11% of the parents of the TD group, 23% of the parents of the EL-no-ASD group and 11% of the parents of the EL-ASD group reported being born in a country other than Sweden. Socioeconomic status was assessed based on family income and parental education level, and did not differ between groups. All infants were born full term (>36 weeks) and infants with confirmed or suspected medical problems, including visual/auditory impairment, were not included. Developmental level was assessed at each visit using the Mullen Scales of Early Learning (MSEL; Mullen, 1995). While developmental level at 10 months did not differ significantly between groups, developmental level at 14 and 18 months was lower in the EL-ASD-group, which is to be expected considering that around 30% of all children with ASD also meet diagnostic criteria for intellectual disability (ID; Maenner et al., 2020). However, mean developmental level for all groups was well above the ID range. All infants went through a comprehensive clinical assessment at 36 months, performed by experienced clinicians and comprising the Autism Diagnostic Observation Schedule, second edition (ADOS-2; Lord et al., 2012) and the Autism Diagnostic Interview—Revised (Rutter et al., 2003). Diagnostic classification was based on *DSM*-5 criteria. Four children fulfilled diagnostic criteria for ASD symptom-wise (and are classified as EL-ASD in the main analyses and in Table 1), but it was not possible to fully verify if *DSM*-5 criterion D (clinical impairment) was fulfilled at the time of assessment. Therefore, sensitivity analyses were run to check whether the overall pattern of results changed depending on the inclusion of these participants (overall patterns did not change, but see results at the end of the results section). Written informed consent was provided by all parents, and the study was approved by the Ethics Board in Stockholm and conducted in accordance with the 1964 Declaration of Helsinki.

**Table 1.** Participant characteristics.

	TD (total *N* = 29; 15 boys)	EL-no-ASD (total *N* = 72; 35 boys)	EL-ASD (total *N* = 25; 15 boys)	*p* value
10 months, *N*	22 (11 boys)	51^1^ (24 boys)	13 (5 boys)	.798^2^
14 months, *N*	25^3^ (13 boys)	58 (28 boys)	21 (12 boys)	.779^2^
18 months, *N*	24 (12 boys)	50^4^ (22 boys)	21 (13 boys)	.387^2^
Age 10 months, M (*SD*)	10.18 (0.45)	10.30 (0.47)	10.26 (0.53)	.532^5^
Age 14 months, M (*SD*)	14.35 (0.64)	14.17 (0.53)	14.33 (0.41)	.253^5^
Age 18 months, M (*SD*)	18.43 (1.04)	18.29 (0.54)	18.33 (0.50)	.675^5^
MSEL 10 months, M (*SD*)	103.79 (12.49)	102.19 (12.57)	98.00 (14.50)	.239^5^
MSEL 14 months, M (*SD*)	97.07 (12.64)	97.74 (10.56)	91.21 (10.99)	.046^5^
MSEL 18 months, M (*SD*)	99.08 (13.41)	98.10 (15.87)	89.04 (12.90)	.024^5^
SES^6^, M (*SD*), *N*	0.20 (0.79)	–0.02 (0.81)	–0.28 (0.83)	.099^5^

TD: typically developing; EL-ASD: elevated likelihood of ASD; MSEL: Mullen Scales of Early Learning; SES: Socioeconomic status.

1 for accuracy analysis, *N* = 52; ^2^Pearson chi-square test comparing the gender ratio between groups; ^3^for latency analysis, *N* = 24; ^4^for latency analysis, *N* = 48; ^5^one-way ANOVA; ^6^Socioeconomic status calculated on the basis of parental education and income (equal weighing), expressed as a *z*-score; for this analysis *N* = 28 in the TD group and 68 in the EL-no-ASD group, as five families did not disclose this information.

### Procedure and stimuli

**Figure 1.**
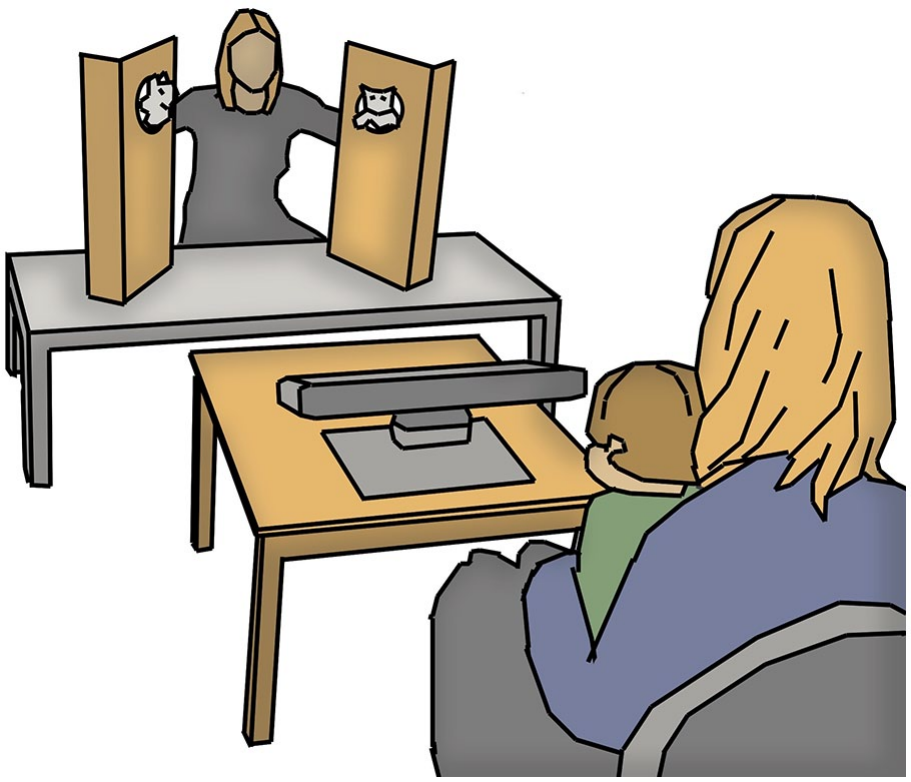
Sketch of the experimental setting. Infant and parent were seated at 200 cm from the experimenter and stimulus area. Placed on the table was a TobiTX300 eye tracker that recorded the gaze of the infant. Two video cameras, not visible in sketch, recorded the infant and the stimulus area.

The gaze following experiment was embedded in a puppet show, lasting approximately 8–10 minutes in total and comprising other experiments as well. Only gaze following tasks will be described here, for reports on other tasks, see Nyström et al. (2017); Nyström et al. (2019). The infant was seated on the lap of a parent, at a distance of 200 cm from the model. The model was seated at a low table, with two wooden screens—each with a hole in it—mounted on top of it. The infant’s gaze was recorded by a Tobii TX300 eye tracker placed in front of the infant, and two video cameras were used to record the behavior of the infant as well as the stimulus area (Figure 1). Before the session started, a 5-point calibration procedure was conducted and if necessary repeated until satisfactory calibration was achieved.

The gaze following trials comprised of four blocks, with other tasks in between. Each block started by the experimenter making two puppets appear through the holes in the wooden screens, keeping hands and arms hidden behind the screens. The puppets appeared at a distance of 25–30 cm from the model’s face. At the start of each trial, the model called the infant’s name to attract its attention to the model’s face. If necessary, the name was called a second time, and if the infant still did not respond, the model made a third attempt by making a funny face and a sound. Once the infant looked at the model’s face, the model shifted gaze toward one of the puppets (or, in the Eyes and Head-No Object condition, to the hole in the screen) while making an excited vocalization (“Oj!” a Swedish interjection expressing surprise or excitement). The model kept looking at the puppet or hole for 4 s before the trial was ended. Each block consisted of six trials, belonging to three conditions (in a few cases more trials were included by mistake and data from these were also included in the analysis). In the Eyes and Head-Object Present condition, the model turned his or her entire head in the direction of the puppet. In the Eyes Only condition, the model only used his or her eyes to gaze at the puppet, while keeping the head facing forward. The Eyes and Head-Object Present and Eyes Only conditions were presented sequentially within blocks, and counterbalanced across blocks. After the four trials belonging to these two conditions had been administered, the model made the puppets disappear under the table. Then, the two Eyes and Head-No Object trials were presented. These trials were identical to the Eyes and Head-Object Present trials in all aspects except for the fact that the puppets no longer were visible. That is, the model turned the head to look at the empty holes in the screens. As the experiment was conducted live, it was performed by several models. To prevent individual differences in interaction style to influence the results, the session was highly standardized. All new models were trained to follow a script, using a video template of the whole session. The same person (T.F-Y) supervised training of all models and ensured that they satisfactorily adhered to the script before they proceeded to conducting the experiment.

### Analysis

**Figure 2.**
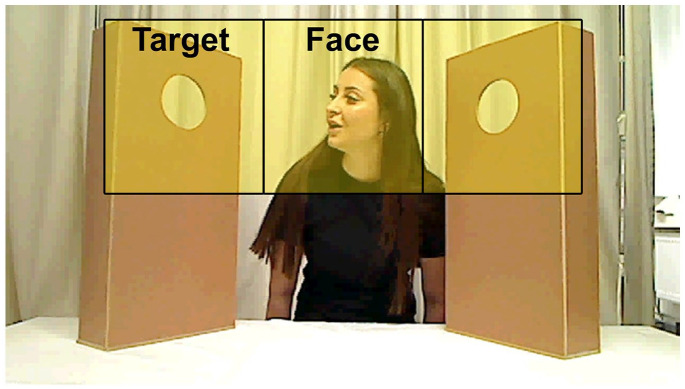
Image of the stimulus area (in the Eyes and Head-No Object condition) with AOIs superimposed. The visual angle of each AOI subtended 10.9° by 9.31°.

Data preparation was performed with MATLAB (The MathWorks, Inc., Natick, MA) using the TimeStudio analysis framework (Nyström et al., 2016). Both raw gaze data and fixation filtered data (Tobii Fixation Filter with default settings) were extracted from the eye tracker data files. The raw data were used to define AOIs and visually assess gaze data quality, and the fixation filtered data were used to extract gaze positions and calculate looking times, as explained below. Data were extracted from four areas of interests (AOIs): one covering the face of the model, two covering the holes in the screens where the puppets appeared, and one covering the entire stimulus area (see Figure 2). To define the AOIs, histograms of the raw gaze points’ position during the task were plotted. As expected by our scene design, the histograms typically showed three well-defined peaks in the *x*-dimension and one in the *y*-dimension. In order to separate the AOIs maximally from each other, we defined the experimenter AOI bounds by the local minima between the three peaks in the *x*-dimension, and used a predefined height that was centered over the peak in the y-dimension. Gaze data were then plotted together with the AOIs so that we could visually inspect the positions of the AOIs and manually reject trials where the fixation classification did not harmonize with the raw data, when data were missing in important time intervals, or when data contained artifacts or excessive noise in important time intervals. Visual inspection was conducted blind to the group status of the infants. Trials with <50% gaze data were excluded automatically. The remaining trials were visually inspected by plotting the gaze coordinates (*x, y*) over time together with AOI positions. Based on these plots, two independent raters (E.T. and P.N.) rated all trials as either valid or invalid (Cohen’s kappa = 0.85). To be included in the analysis, each infant had to contribute at least 25% valid trials, that is, a total of six valid trials, and at least one valid trial in each condition, at each age (for comparisons of the number of valid trials, see Table 2). For more details concerning data preparation, see Nyström et al. (2019).

**Table 2.** Number of valid trials per assessment point and measure.

	TD	EL-no-ASD	EL-ASD	*p* value^1^
Accuracy, 10 months, M (*SD*)	16.36 (4.50)	14.83 (4.57)	14.23 (4.64)	.315
Accuracy, 14 months, M (*SD*)	16.92 (4.74)	16.02 (4.19)	14.62 (4.25)	.203
Accuracy, 18 months, M (*SD*)	16.42 (3.98)	16.02 (3.88)	15.33 (4.05)	.651
Looking durations, 10 months, M (*SD*)	11.50 (4.61)	10.53 (4.03)	9.23 (2.95)	.280
Looking durations, 14 months, M (*SD*)	14.40 (5.02)	12.60 (4.53)	11.48 (3.54)	.082
Looking durations, 18 months, M (*SD*)	13.33 (3.78)	12.94 (3.79)	11.86 (3.68)	.396
Latency, 10 months, M (*SD*)	9.18 (3.98)	8.25 (3.52)	7.46 (2.93)	.365
Latency, 14 months, M (*SD*)	11.76 (5.10)	10.31 (4.40)	9.15 (3.79)	.140
Latency, 18 months, M (*SD*)	10.83 (4.17)	10.48 (3.93)	9.67 (4.13)	.611

TD: typically developing; EL-ASD: elevated likelihood of ASD.

1 One-way ANOVA.

Statistical analyses were performed in SPSS. For the accuracy analysis, the dependent measure was the percentage of trials were the infant followed gaze (i.e. looked at the attended target area first), out of the total number of trials where the infant either did or did not follow gaze (i.e. looked at the unattended area first; trials in which the infant did not look at either the attended or unattended area were not included). We chose to use a proportional measure rather than a difference score as in our previous study (Nyström et al., 2019), as a standardized measure is less affected by possible group differences in the number of valid trials.

As our major interest was on gaze behaviors occurring in conjunction with successful gaze following, all remaining analyses were performed only on those trials where the infants *did* follow gaze. For the looking duration analyses, the percentage of time spent looking at the target area as well as the model’s face, out of the total time spent looking anywhere at the stimulus area (including the unattended object), were chosen as dependent measures. Looking time was measured from when the infant’s gaze first landed on the face AOI (after the model had started looking toward the target area) and until the end of trial. As a measure of how fast the infants would look back at the model after following gaze, the latency by which gaze reached the model’s face after landing on the target area was used. All dependent measures were averaged across trials (per age and condition), and statistical analysis was performed on the mean values.

For each dependent measure, a linear mixed model with the restricted maximum likelihood method was conducted. Linear mixed models were chosen as they allow individuals to be included in the analysis even if they do not contribute data at all three measurement points. Condition (Eyes and Head-Object Present, Eyes Only or Eyes and Head-No Object), age (10, 14, or 18 months) and group (EL-ASD, EL-no-ASD or TD) were entered as fixed factors and subject was entered as a random factor (with intercept allowed to vary between participants). After running initial models, non-significant interaction terms were removed. Significant main effects were followed-up using Bonferroni-corrected pairwise comparisons on the estimated marginal means.

There is no community involvement in this study.

## Results

### Accuracy

Please note that accuracy results partly represent a re-analysis of data that have been reported on earlier (age effects and comparisons between Eyes and Head-Object Present and Eyes Only conditions are reported by Nyström et al., 2019 and will thus not be further discussed in this paper), and are reported here primarily for completeness in relation to the subsequent analyses.

**Figure 3.**
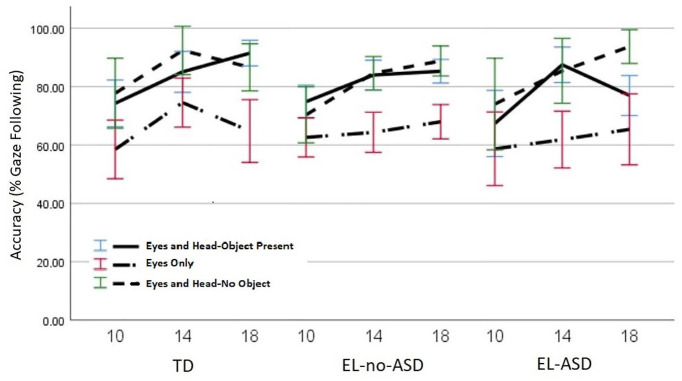
Gaze following accuracy expressed at the percentage of trials where infants looked at the attended target area first (out of all trials with gaze data at either attended or unattended target area). Performance is reported at 10, 14, and 18 months. Error bars represent standard errors.

A linear mixed model with accuracy as outcome variable and age, condition and group as predictors revealed significant main effects for age, *F*(2, 834.16) = 23.11, *p* < .001 and condition, *F*(2, 738.95) = 64.58, *p* < .001, but no significant effect of group, *F*(2, 117.59) = 1.21, *p* = .303, and no significant interaction effects (see Figure 3). Gaze following accuracy increased between 10 (M = 68.97%, *SE* = 1.53, 95% CI (65.95, 71.98)) and 14 months (M = 79.50%, *SE* = 1.40, 95% CI (76.75, 82.26)), *p* < .001, but did not differ significantly between 14 and 18 months (M = 80.60%, *SE* = 1.44, 95% CI (77.77, 83.43)), *p* = 1.00. Gaze following accuracy was lower in the Eyes Only condition (M = 64.60%, *SE* = 1.44, 95% CI (61.77, 67.44)) compared to both the Eyes and Head-Object Present condition (M = 81.33%, *SE* = 1.44, 95% CI (78.50, 84.179), *p* < .001, and the Eyes and Head-No Object condition (M = 83.13%, *SE* = 1.44, 95% CI (80.30, 85.97)), *p* < .001. Gaze following accuracy did not differ between the Eyes and Head-Object Present and Eyes and Head-No Object conditions, *p* = .952.

### Looking duration measures

**Figure 4.**
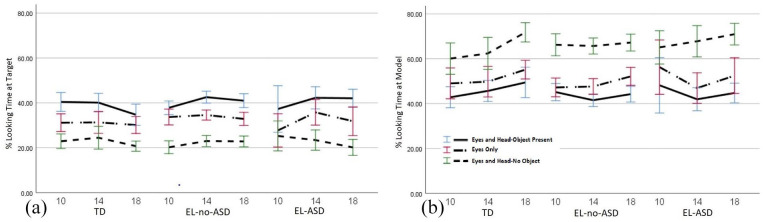
Percentage of time that the infants spent looking at the target (a) and model (b), out of the total time spent looking anywhere on the stimulus area. Performance is reported at 10, 14, and 18 months. Error bars represent standard errors.

A linear mixed model with looking duration at the target area as outcome variable and age, condition and group as predictors revealed significant main effects for age, *F*(2, 849.38) = 5.28, *p* = .002; and condition, *F*(2, 776.19) = 215.72, *p* < .001, but no main effect for group, *F*(2, 115.01) = 0.68, *p* = .508 and no interaction effects (see Figure 4(a)). Looking duration at the target area increased between 10 months (M = 30.44%, *SE* = 0.73, 95% CI (29.01, 31.87)) and 14 months (M = 33.19%, *SE* = 0.69, 95% CI (31.84, 34.54)), *p* = .005, and then decreased again between 14 months and 18 months (M = 30.86%, *SE* = 0.69, 95% CI (29.50, 32.22)), *p* = .017. Infants looked longer at the target area in the Eyes and Head-Object Present condition (M = 39.79%, *SE* = 0.68, 95% CI (38.45, 41.13)) compared to both the Eyes Only condition (M = 32.50%, *SE* = 0.70, 95% CI (31.13, 33.87)), *p* < .001, and the Eyes and Head-No Object condition (M = 22.20%, *SE* = 0.71, 95% CI (20.81, 23.60)), *p* < .001. They also looked longer at the target area in the Eyes Only condition compared to the Eyes and Head-No Object condition, *p* < .001.

A linear mixed model with looking duration at the model’s face as outcome variable and age, condition and group as predictors revealed significant main effects for age, *F*(2, 833.61) = 8.51, *p* < .001; and condition, *F*(2, 770.95) = 209.54, *p* < .001, but no main effect for group, *F*(2, 118.46) = 0.11, *p* = .896 and no interaction effects (see Figure 4(b)). 18-month olds (M = 56.13%, *SE* = 1.02, 95% CI (54.13, 58.13)) looked longer at the model compared to 14-month olds (M = 51.77, %, *SE* = 1.01, 95% CI (49.79, 53.75)), *p* < .001, and 10-month olds (M = 53.00%, *SE* = 1.06, 95% CI (50.92, 55.08)), *p* = .018. There was no difference between the looking durations of 10- and 14-month olds, *p* = .818. Infants looked longer at the model in the Eyes and Head-No Object condition (M = 66.17%, *SE* = 1.03, 95% CI (64.14, 68.20)) compared to both the Eyes and Head-Object Present condition (M = 44.74%, *SE* = 1.00, 95% CI (42.77, 46.71)), *p* < .001, and the Eyes Only condition (M = 49.99%, *SE* = 1.01, 95% CI (47.99, 51.99), *p* < .001. They also looked longer at the model in the Eyes Only condition compared to the Eyes and Head-Object Present condition, *p* < .001.

### Latency to look back at model’s face

**Figure 5.**
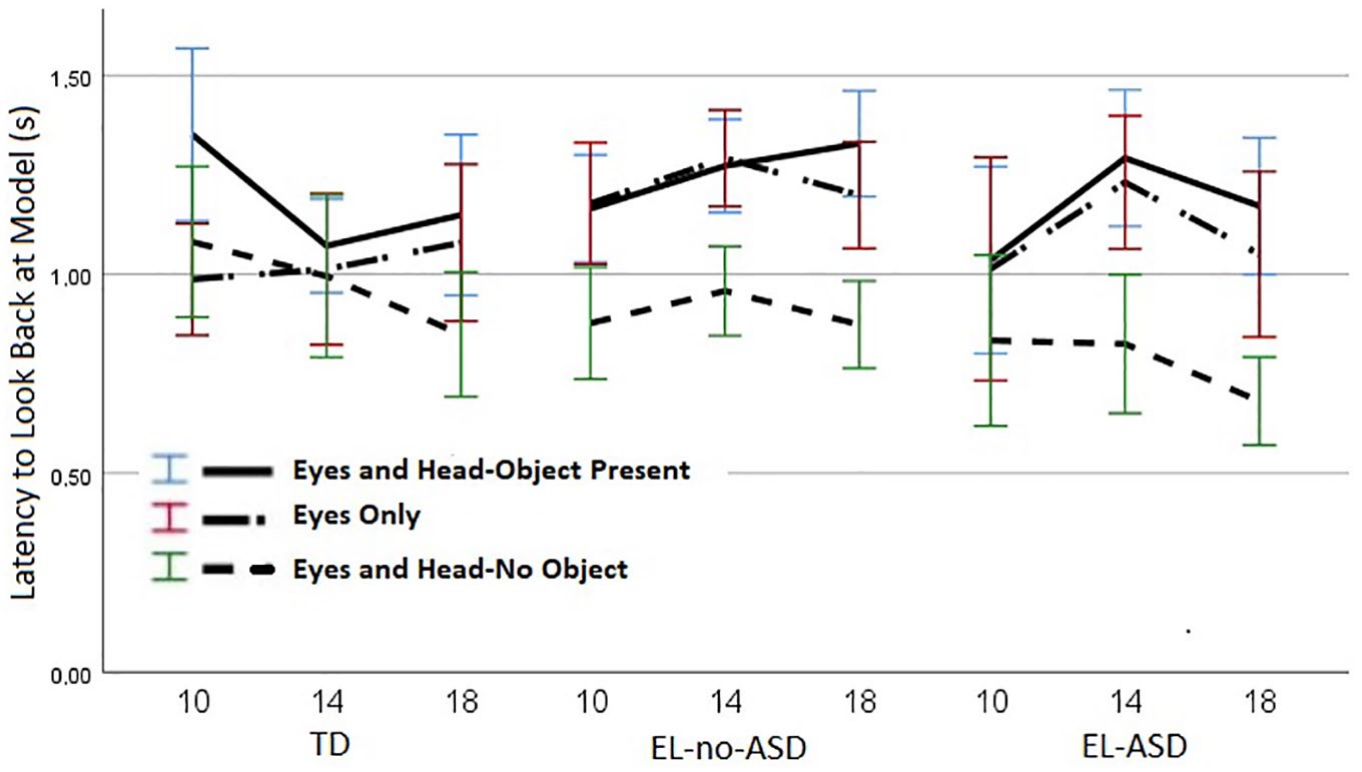
Latency(s) by which gaze reached the model after landing on the target area. Performance is reported at 10, 14, and 18 months. Error bars represent standard errors.

A linear mixed model with latency as outcome variable and age, condition and group as predictors revealed a significant main effect of condition, *F*(2, 732.91) = 36.12, *p* < .001, and a significant interaction between condition and group, *F*(4, 733.08) = 2.70, *p* = .030. There were no significant effects of age *F*(2, 801.71) = 1.79, *p* = .167, or group, *F*(2, 116.63) = 2.45, *p* = .091 and no other interaction effects (see Figure 5). Latencies were shorter in the Eyes and Head-No Object condition (M = 0.88 s, *SE* = 0.03, 95% CI (0.81, 0.94)) compared to both the Eyes and Head-Object Present condition (M = 1.21 s, *SE* = 0.03, 95% CI (1.15, 1,27)), *p* < 0.001, and the Eyes Only condition (M = 1.12 s, *SE* = 0.03, 95% CI (1.05, 1.18)), *p* < .001. Latencies did not differ between the Eyes and Head-Object Present condition and the Eyes Only condition, *p* = .059.

In order to follow-up on the interaction between condition and group, a linear mixed model with group as the only fixed factor was conducted in each condition separately. The analysis revealed a significant effect of group in the Eyes and Head-No Object condition, *F*(2, 113.27) = 3.16, *p* = .046. In this condition, the EL-ASD group displayed shorter latencies from target back to face (M = 0.77 s, *SE* = 0.06, 95% CI (0.65, 0.89) compared to the TD group (M = 0.97 s, *SE* = 0.05, 95% CI (0.86, 1.07)), *p* = .046. There was no difference between the latencies of the EL-ASD and the EL-no-ASD group (M = 0.91 s, *SE* = 0.04, 95% CI (0.84, 0.98)), *p* = 0.149, and also no difference between the EL-no-ASD and the TD group, *p* = 1.00. There was also a significant effect of group in the Eyes Only condition, *F*(2, 109.96) = 4.03, *p* = .021. Here, the EL-no-ASD group displayed longer latencies (M = 1.23 s, *SE* = 0.04 s, 95% CI (1.15, 1.30)) compared to the TD group (M = 1.03 s, *SE* = 0.06, 95% CI (0.91, 1.15)), *p* = .024. There was no difference between the latencies of the TD group and the EL-ASD group (M = 1.10 s, *SE* = 0.07 s, 0.97, 1.24)), *p* = 1.00, and also no differences between the latencies of the two EL groups, *p* = .352. In the Eyes and Head-Object Present condition, there was no significant effect of group, *F*(2, 120.22) = 0.81, *p* = .448.

### Sensitivity analyses

In order to check whether the inclusion of the four children who had been classified as EL-ASD but with uncertainty regarding the *DSM*-5 criteria D (see Methods) affected the results, all analyses were re-run (1) excluding these children from the sample and (2) reclassifying these children as EL-no-ASD. All major patterns of results remained similar. In the latency analysis, a group by age interaction emerged both when excluding, *F*(4, 766.21) = 2.76, *p* = .027 and reclassifying the children, *F*(4, 790.81) = 2.68, *p* = .031. However, following up this interaction effect did not reveal any significant differences between groups at any age.

## Discussion

### Shorter latencies to look back in the EL-ASD group in the absence of target objects

Contrary to our hypothesis, we did not observe shorter looking durations at the target area in the Eyes and Head-No Object condition in the infants with later ASD. Instead, a group by condition interaction effect emerged in the latency analysis, indicating that infants with a later ASD diagnosis were faster to look back at the model compared to TD infants in the Eyes and Head-No Object condition but not in the Eyes and Head-Object Present condition (which was identical to the Eyes and Head-No Object condition except for that target objects were present).

Although this effect was not expected a priori, it may reflect the same mechanism that we hypothesized to result in shorter total looking time at target areas without present objects in the EL-ASD group. It is possible that infants with later ASD look away from the empty hole in the Eyes and Head-No Object condition faster because seeing another person look at it does not result in an attentional heightening to the same extent as in TD infants (Csibra & Volein, 2008). In other words, to the infants in the EL-ASD group, the target area may represent an empty hole, whereas it for TD infants may represent an empty hole *that has caught someone else’s attention*. This interpretation is in line with the previous suggestion that other’s gaze may influence scene and object processing more in TD than in ASD (Falck-Ytter et al., 2015; Freeth et al., 2010; Thorup et al., 2017). Alternatively, quickly looking back at the interlocutor’s face after establishing that he or she is looking at an empty hole could reflect an information seeking strategy, or what is typically referred to as social referencing, that is, infants’ tendency to look at another person for guidance when faced with an ambiguous situation (e.g. Stenberg & Hagekull, 1997). However, in that case, the results would contradict previous findings of *slower* social referencing (Cornew et al., 2012) as well as a lower tendency to engage in social referencing altogether (Gammer et al., 2015) in infants later diagnosed with ASD.

It should be noted that there was no significant difference between the latencies of the EL-ASD and the EL-no-ASD groups, which suggests that although the latency measure can distinguish between infants with later ASD and TD infants, it did not differentiate ASD versus non-ASD within an EL sample. However, we note that the EL-no-ASD group’s latency scores fell in between the other two groups, which fits with the fact that this group includes a substantial number of infants with elevated ASD symptoms, as well as symptoms of other related conditions (Ozonoff et al., 2014; Shephard et al., 2017).

Unexpectedly, longer latencies were detected in the EL-no-ASD group compared to the TD group (with the EL-ASD group falling in between) in the Eyes Only condition specifically. We cannot think of any theoretical reason for why the more diverse EL-no-ASD group, but not the EL-ASD group, should be differently affected than the TD group in this condition. It is possible that the finding represents a spurious relationship. We therefore refrain from further interpretation at this stage but recommend that future studies investigate whether the finding is replicable.

### No group differences in total looking time at model’s face and target area

Infants with later ASD did not differ from other infants in terms of the total time they spent looking at the model’s face or the target area, and excluding target objects did not affect their looking durations at targets or the face differently compared to the other groups of infants. Previous studies have come to inconsistent conclusions regarding whether children with (concurrent or later) ASD differ from TD children in terms of attention allocation while the model is attentionally engaged with the target. Some studies report less looking at the model in ASD (Chawarska et al., 2013; Vivanti et al., 2017) and others report similar looking times across groups (Billeci et al., 2016; Chawarska et al., 2012; Parsons et al., 2019). Similarly, a number of studies report less looking at the target object in ASD (Bedford et al., 2012; Parsons et al., 2019; Vivanti et al., 2017) and others report no group differences (Bedford et al., 2012; Billeci et al., 2016; Gliga et al., 2012; Parsons et al., 2019). Note that two of the studies are cited twice: Bedford et al. (2012) reported less looking at the target in ASD at 13 months, but not at 7 months; Parsons et al. (2019) found reduced looking in ASD when comparing looking time at target to total looking time at screen, but no difference when comparing looking at target to looking at non-target. Although differences in methodology and age groups make direct comparisons between studies difficult, the current results add to the majority, thus strengthening the view that in terms of looking time to face and target, there are no striking differences between children with ASD/infants at elevated likelihood of ASD and other children. When comparing results across studies, it should be emphasized that this study is the only one using a live paradigm. Although screen-based eye tracking entail better control and more options for manipulation, it could be argued that live set-ups may be more suitable when the measured behaviors are of a social nature. Studies have shown discrepancies in how humans look at people live versus on video (Foulsham et al., 2011; Laidlaw et al., 2011). Engaging in interaction has also been shown to recruit brain areas associated with social cognition to a higher degree than watching prerecorded interactions (Redcay et al., 2010). We therefore suggest that our live paradigm is more likely to engage “the social brain” and capture behaviors as they occur in “real life” compared to previous work with prerecorded stimuli.

### No group differences in gaze following accuracy

Eliminating objects from the target area did not affect gaze following accuracy differently in infants with later ASD compared to other infants. That we also found no main effect of group in terms of the accuracy measure is interesting (and in line with much previous research; Akechi et al., 2011; Bedford et al., 2012; Falck-Ytter et al., 2015; Gliga et al., 2012; Parsons et al., 2019), but as it is not a novel finding (it is based on largely the same data as previously reported on) we refer to our previous paper (Nyström et al., 2019) for further discussion. It is noteworthy however, that compared to Nyström et al. (2019), this study used a slightly different operationalization of accuracy, that is, % rather than a difference score, and the results also differ slightly. In the previous study using a difference score (*N* congruent trials minus *N* incongruent trials), we found reduced gaze following accuracy in both EL groups compared to the TD group (but no difference between those EL infants who did receive a later diagnosis and those who did not). When re-analyzing our current data with such a difference score, a similar group effect emerged. However, because the difference score measure is dependent on the number of valid trials, which descriptively is lower in EL-ASD (although the difference is not statistically significant at most ages did not reach statistical significance), we considered it more accurate to use a ratio-based measure in this study.

### General findings concerning gaze cues and targets

Although most analyses did not evoke any (main or interaction) effects pertaining to group status, they all revealed main effects of condition and age. These analyses are therefore informative on how aspects pertaining to the gaze cue versus target affect looking behaviors in typical development, and they also highlight some general developmental trends. Gaze following accuracy was higher in the Eyes and Head-Object Present and Eyes and Head-No Object conditions compared to the Eyes Only condition. This suggests that increasing the saliency of the gaze cue (by using both eye and head movement as opposed to only eye movement) affects gaze following positively, but that increasing the saliency of the target (by presenting as opposed to not presenting objects) does not further improve performance. For gaze following accuracy, aspects pertaining to the cue therefore seem to be more important than those pertaining to the target. This finding has possible implications for interventions for children with JA impairments, and is in line with a previous study, in which we showed that although increasing the interest level of the target objects led to more total looking at the objects, it did not lead to increased gaze following accuracy neither in autistic nor TD children (Thorup et al., 2017). The looking duration analyses revealed a clear pattern with the Eyes and Head-Object Present condition being associated with the highest proportion of looking at target and lowest at model, and the Eyes and Head-No Object condition with the opposite (highest proportion looking at model and lowest at target). The Eyes Only condition fell in between the other two. That infants spent least time looking at the target area in the Eyes and Head-No Object condition is not surprising, as there *is* no target object to look at in this condition. The results further suggest that sustained looking at target objects is facilitated by the use of salient directional cues (both eye direction and head movement). Infants’ looking duration at target objects is thus influenced both by the saliency of the target and by that of the gaze cue. The longitudinal analyses revealed an increase in looking time at the target area between 10 and 14 months, but then a decrease between 14 and 18 months. During this age period, infants instead increased their time spent looking at the model, perhaps indicating a greater social interest with increasing age (Di Giorgio et al., 2012; Frank et al., 2009).

### Limitations and future research

The current study has some notable limitations. While the order of the Eyes and Head-Object Present and Eyes Only conditions was alternated between blocks, the Eyes and Head-No Object condition always appeared last in the block. The reason for this was partly to increase the “narrative” of the puppet show (puppets disappearing after first having been shown within block), but also to minimize the administrative burden of the experimenter (full counterbalancing would increase the difficulty of presentation, which could reduce the quality of the performance). Although the fact that gaze following accuracy was higher in the last occurring Eyes and Head-No Object condition than in the earlier occurring Eyes Only condition suggests that no order effect was present in terms of this particular measure, order effects may have affected other results. It is thus not possible to rule out that group differences in terms of learning strategies and habituation (for a review of altered habituation in ASD, see McDiarmid et al., 2017) may have had an impact on the results. In the Eyes and Head-No Object condition the model did not just look at an empty hole, but at *an empty hole from where a puppet had just disappeared*. Again, we cannot rule out that this “storyline” of puppets appearing and disappearing affected the groups differently. Also, it could be argued that the puppets primed the infants’ attention to the stimulus area. Although perhaps a bit speculative, it is possible that the EL-ASD group may have shown lesser interest in the empty stimulus area had that not been the case. We recommend that future studies investigate gaze following to empty areas without previously appearing stimuli in the same areas.

Finally, the relatively modest sample size of our EL-ASD group must be noted as a weakness, and lack of power considered as a possible explanation for the findings of similar performance across groups on most measures. The fact that we have previously (Nyström et al., 2019) detected group differences in a largely over-lapping sample of similar size and with a similar analytic approach may suggest that we have sufficient power to detect group differences, but replication in a larger sample is nevertheless warranted.

## Conclusion

Taken together, the results of the present study suggest that gaze following is largely typical in infants with later ASD. However, our finding regarding the latency measure suggests that there may be subtle atypicalities in gaze behaviors occurring just *after* gaze following. Previous work on older children have suggested that autistic children may be less affected by others’ looking behaviors when processing visual stimuli (Falck-Ytter et al., 2015; Freeth et al., 2010; Thorup et al., 2017). Although it would be premature to conclude that the current finding of shorter latencies in the No Object condition in the EL-ASD group is a manifestation of this, the finding highlights an interesting area. Future studies may both further investigate whether infants with later ASD are indeed less influenced by other’s gaze, and what effects such a lesser influence may have on learning and development.
